# Biointegrated Conductive Hydrogel for Real-Time Motion Sensing in Exoskeleton-Assisted Lower-Limb Rehabilitation

**DOI:** 10.3390/s25216727

**Published:** 2025-11-03

**Authors:** Ming Li, Hui Li, Yujie Su, Raymond Kai-Yu Tong, Hongliu Yu

**Affiliations:** 1 Institute of Intelligent Rehabilitation Engineering, University of Shanghai for Science and Technology, Shanghai 200093, China; 2 Shanghai Engineering Research Center of Assistive Devices, Shanghai 200093, China; 3 Key Laboratory of Neural-Functional Information and Rehabilitation Engineering of the Ministry of Civil Affairs, Shanghai 200093, China; 4 Department of Biomedical Engineering, The Chinese University of Hong Kong, Hong Kong SAR 999077, China

**Keywords:** wearable sensor, lower-limb exoskeleton, conductive hydrogel, rehabilitation

## Abstract

**Highlights:**

**What are the main findings?**

- A multifunctional CPSD composite hydrogel was developed by integrating conductive PEDOT:PSS and dopamine-functionalized alginate into a carboxymethyl chitosan matrix.
- The hydrogel exhibits strong antioxidant, antibacterial, and biocompatible properties with stable adhesion under dynamic exoskeleton-assisted rehabilitation.

**What are the implications of the main findings?**

- The CPSD hydrogel enables simultaneous wound protection and real-time lower-limb motion sensing.
- This work provides a foundation for closed-loop wearable rehabilitation systems that combine bioelectronic sensing with therapeutic functionality.

**Abstract:**

Chronic lower-extremity wounds in patients undergoing exoskeleton-assisted rehabilitation require materials that can both protect tissue and enable real-time physiological monitoring. Conventional dressings lack dynamic sensing capability, while current conductive hydrogels often compromise either adhesion or electronic performance. Here, we present a biointegrated hydrogel (CPSD) composed of carboxymethyl chitosan (CMCS) and poly(3,4-ethylenedioxythiophene):poly(styrenesulfonate) (PEDOT:PSS) forming the conductive backbone, integrated with dopamine-functionalized sodium alginate (SD); the network is assembled via electrostatic complexation and carbodiimide (EDC/NHS)-mediated covalent crosslinking. The resulting hydrogel exhibits a dense, tissue-conformal porous network with tunable swelling, stable mechanical integrity, and high photothermal conversion efficiency. In vitro assays confirmed potent antioxidant activity, strong antibacterial performance (>90% under near-infrared), and excellent cytocompatibility and hemocompatibility. CPSD shows bulk conductivity ~1.6 S·m^−1^, compressive modulus ~15 kPa, lap-shear adhesion on porcine skin ~9.5 kPa, and WVTR ~75 g·m^−2^·h^−1^, supporting stable biointerfaces for motion/sEMG sensing. Integrated into a lower-limb exoskeleton, CPSD hydrogels adhered securely during motion and reliably captured electromyographic and strain signals, enabling movement-intent detection. These results highlight CPSD hydrogel as a multifunctional interface material for next-generation closed-loop rehabilitation systems and mobile health monitoring.

## 1. Introduction

Lower-limb exoskeleton robots have been shown to improve mobility in individuals with lower-extremity functional impairments [1,2,3]. Achieving effective human–robot coordination [4,5,6], however, requires precise recognition of the user’s movement intent—often relying on integrated sensing modalities such as electromyography (EMG) [7,8,9] and mechanical force sensors [10,11,12]. In parallel, chronic lower-extremity wounds are becoming an increasingly significant healthcare challenge [13], particularly for patients undergoing exoskeleton-assisted rehabilitation [14]. While conventional wound dressings can provide passive protection [15], they lack the capability to dynamically track healing progress—a critical limitation, as improper loading during mobility can markedly impede recovery [16,17,18].

The integration of flexible sensors into rehabilitative devices has emerged as a promising solution [19,20,21], yet the development of materials that can simultaneously conform to moving anatomical structures [22], resist biofouling, and maintain sensing accuracy remains an unmet need [23]. Recent advances in conductive hydrogels have demonstrated tissue-like mechanical compliance and promising potential for biomedical sensing [24,25]. In particular, systems combining poly(3,4-ethylenedioxythiophene): poly(3,4-ethylenedioxythiophene):poly(styrenesulfonate) (PEDOT:PSS) with chitosan derivatives offer inherent antibacterial activity to prevent infection, together with sufficient conductivity for physiological signal acquisition [26,27]. Nevertheless, these materials often fail to maintain stable skin adhesion during prolonged wear, especially in the presence of wound exudate [28]. Mussel-inspired adhesive hydrogels can address adhesion challenges but typically do so at the expense of electronic performance—a fundamental trade-off that limits their utility in active monitoring applications [29].

Furthermore, near-infrared (NIR) irradiation was incorporated as an auxiliary stimulus to enhance the antibacterial efficiency of the hydrogel while maintaining biosafety. Catechol and π-conjugated PEDOT:PSS domains can convert NIR light into localized heat through non-radiative relaxation, producing mild photothermal effects that disrupt bacterial membranes and improve ROS-scavenging balance within moist wounds [30]. Such NIR-responsive behavior has been widely explored for synergistic disinfection and accelerated tissue repair in polymeric hydrogels [31]. This mechanistic rationale justifies the inclusion of NIR irradiation in our design, aligning it with the dual therapeutic and sensing functions required for exoskeleton-assisted rehabilitation [32].

This work targets a biointegrated interface that concurrently protects moist wound beds and enables real-time motion/sEMG sensing during exoskeleton-assisted lower-limb rehabilitation. Conventional gelled Ag/AgCl or dry electrodes often suffer from motion artifacts and adhesion loss on perspiring skin and dynamic joints, while purely adhesive hydrogels typically lack robust electronic coupling and benchmarking [33]. We therefore design a CPSD hydrogel that couples robust wet-tissue adhesion, breathable moisture management (WVTR), ROS-scavenging/antibacterial microenvironment regulation, and biopotential acquisition benchmarked against Ag/AgCl, with agreement quantified at the PSD level by Bland–Altman [34].

Here, we present a biointegrated hydrogel platform that reconciles these competing requirements through rational material hybridization [35]. By combining a chitosan–PEDOT:PSS conductive network with dopamine-functionalized alginate adhesives, we developed a composite system (CPSD hydrogel) capable of both protecting wounds and intelligently responding to tissue status [36]. We demonstrate that CPSD hydrogels can monitor strain patterns during exoskeleton-assisted movement. The design principles established herein may be extended to other mobile health monitoring applications requiring robust, long-term biointerfaces.

## 2. Materials and Methods

### 2.1. Materials

Carboxymethyl chitosan (CMCS, Mw ~20–30 kDa, degree of deacetylation ≥ 90%, degree of substitution ≥ 90%) was purchased from Shanghai Macklin Biochemical Co., Ltd. (Shanghai, China). PEDOT:PSS, 1.3 wt% dispersion in water and dopamine hydrochloride (DA, 98%) were obtained from Sigma-Aldrich (St. Louis, MO, USA). Sodium alginate (SA, viscosity: 200 ± 20 mPa·s, M/G ratio ≈ 1.5) was supplied by Aladdin Biochemical Technology Co., Ltd. (Shanghai, China). Deionized water (DI water, 18.2 MΩ·cm) was used for all experiments. Cell Counting Kit-8 (CCK-8, Beyotime, Shanghai, China) and Calcein-AM/Propidium Iodide (PI) Live/Dead Cell Staining Kit (Beyotime, Shanghai, China). Dulbecco’s Modified Eagle Medium (DMEM, high glucose, Solarbio, Beijing, China) supplemented with 10% fetal bovine serum (FBS, Solarbio, Beijing, China) and 1% penicillin–streptomycin (100 U·mL^−1^/100 μg·mL^−1^, BasalMedia, Shanghai, China) served as the cell-culture medium. Luria–Bertani (LB) broth was self-prepared per 250 mL batch using tryptone 2.5 g, NaCl 2.5 g, and yeast extract 1.25 g; the LB agar used for solid-phase tests contained the same composition plus agar 4.0 g. All experiments were conducted in accordance with the guidelines approved by the Medical Ethics Committee of Xinhua Hospital, Shanghai Jiao Tong University School of Medicine (Approval No. XHEC-C-2024-202-1).

### 2.2. Preparation and Characterization of Composite Hydrogels

#### 2.2.1. Preparation of CP and SD Nanoparticles

First dissolving CMCS powder in deionized water at 2 wt% concentration with magnetic stirring at 500 rpm for 2 h at 25 °C, followed by adjusting the solution pH to 4.0 using 0.1 M HCl. The PEDOT:PSS aqueous dispersion (1.3 wt%) was then diluted with an equal volume of deionized water and sonicated for 15 min before being slowly added into the CMCS solution at a 1:1 volume ratio under continuous stirring at 800 rpm. The mixture was maintained at 25 °C with stirring for 6 h, after which the resulting composite solution (CP) was rinsed three times with deionized water to remove unbound components.

The DA-grafted SA was prepared by dissolving SA (2.0 g) in deionized water (100 mL) with stirring at 500 rpm for 2 h at 25 °C. DA hydrochloride (0.2 g) and EDC (0.3 g) were added sequentially, followed by NHS (0.15 g). The reaction proceeded at 25 °C for 24 h with stirring. The product was dialyzed (MW, 3500 Da) against deionized water for 48 h and lyophilized to obtain the final product (SD).

#### 2.2.2. Preparation of CMCS/SA Hydrogels

Preparation of CMCS/SA control hydrogels. CMCS powder was dissolved in deionized water to obtain a 2 wt% solution (magnetic stirring, 500 rpm, 2 h, 25 °C), and the pH was adjusted to 4.0 with 0.1 M HCl, following the same procedure as used for CMCS in CPSD precursors. Separately, sodium alginate (SA) was dissolved in deionized water to 2 wt% (500 rpm, 2 h, 25 °C). Equal volumes of CMCS (2 wt%) and SA (2 wt%) were mixed thoroughly, after which EDC (50 mg) and NHS (30 mg) were added as carbodiimide crosslinkers to promote amide bond formation between SA carboxylates and CMCS amines (dosage and curing conditions matched to CPSD). The mixture was homogenized for 30 min at 25 °C, cast into molds, and incubated at 37 °C for 2 h to complete gelation; hydrogels were then rinsed three times with deionized water prior to testing.

#### 2.2.3. Preparation of CPSD Hydrogels

The CPSD hydrogels were prepared by mixing CP solution (2 wt%) with SD solution (1 wt%) at a 1:1 volume ratio. EDC (50 mg) and NHS (30 mg) were added as crosslinkers under stirring at 25 °C. After 30 min of homogenization, the mixture was transferred to molds and incubated at 37 °C for 2 h to complete gelation. Amide coupling between SA-DA and CMCS was induced by EDC/NHS chemistry. The obtained hydrogels were thoroughly rinsed with deionized water to remove residual reagents.

#### 2.2.4. Characterization of CPSD Hydrogels

The CPSD hydrogels were characterized using comprehensive analytical techniques: Morphological analysis was performed by field-emission scanning electron microscopy (FE-SEM, SU8010, Hitachi, Tokyo, Japan) with freeze-dried samples sputter-coated with 5 nm gold and imaged at 5 kV acceleration voltage, while pore size distribution was quantified using ImageJ software (National Institutes of Health, Bethesda, MD, USA; *n* = 50 measurements). Chemical structure was analyzed by Fourier-transform infrared spectroscopy (FTIR, Nicolet iS50, Thermo Fisher Scientific, Waltham, MA, USA) in attenuated total reflection mode (4000–500 cm^−1^ range, 4 cm^−1^ resolution, 32 scans) with second-derivative spectra processed using OMNIC software (Thermo Fisher Scientific, Waltham, MA, USA). Rheological properties were measured using a rotational rheometer (MCR302, Anton Paar, Graz, Austria) equipped with 25 mm parallel plates, conducting frequency sweeps (0.1–100 rad/s at 1% strain) and strain sweeps (0.1–1000% at 1 Hz) at 37 °C to determine storage (G′) and loss (G″) moduli. Optical characterization included UV-Vis absorption spectra (200–800 nm, 1 nm resolution) acquired on a Shimadzu UV-2600 spectrophotometer (Shimadzu Corporation, Nakagyo-ku, Kyoto, Japan) and fluorescence imaging performed with a Nikon A1R confocal microscope (Nikon Corporation, Minato-ku, Tokyo, Japan) using 405/488/561 nm excitation lasers.

Compression of CPSD hydrogels was performed on a universal testing machine (Instron 5943, Instron, Norwood, MA, USA) equipped with a 500 N load cell. Cylindrical specimens (diameter 8–10 mm, height 6–8 mm) were cut from freshly prepared gels, gently blotted to remove excess surface water, and tested in the hydrated state at 37 °C. The compressive modulus was obtained from the linear fit of the initial stress–strain region (typically 0–10% strain). Data are reported as mean ± SD (n = 3).

Bulk conductivity was measured on hydrated slabs using a four-probe resistivity tester (ST-2258C, Suzhou Jingge, Suzhou, China). Samples (thickness t measured with a digital micrometer, ±0.01 mm) were equilibrated at 37 °C in PBS for 30 min, wiped to remove surface liquid, and placed on the stage. The instrument recorded sheet resistance R_s_ (Ω/sq) using a constant-current method with fixed probe spacing. Each value represents mean ± SD (n = 3).

### 2.3. Swelling and Degradation of Composite Hydrogels

The swelling behavior of CPSD hydrogels was examined in both PBS (pH 7.4) and deionized water. Pre-weighed dried hydrogels (W_0_) were immersed in 50 mL of each medium at 37 °C with shaking (100 rpm). Samples were removed at specified time points (1, 2, 4, 8, 12, 24 h), surface-blotted, and weighed (W_t_) to calculate swelling ratio:

(1) $$Swelling\;ratio\;\left(\%\right)=\;\frac{\left(W_{t}-W_{0}\right)}{W_{0}}\;\times 100$$

Degradation studies were conducted in PBS containing 10 U/mL lysozyme and in deionized water. Hydrogels (*W*_0_) were incubated in 50 mL media at 37 °C with shaking (50 rpm). At predetermined intervals (1, 3, 7, 14 d), samples were collected, rinsed, lyophilized, and weighed (*W*_n_) to determine remaining mass:

(2) $$Remaining\;mass\;(\%)=\;\frac{W_{n}}{W_{0}}\;\times 100$$

All experiments were performed in triplicate (n = 3). Data are presented as mean ± standard deviation. Statistical analysis was performed using one-way ANOVA with Tukey’s test (*p* < 0.05).

### 2.4. In Vitro Antibacterial Performance of CPSD Hydrogels

The antibacterial properties of CPSD hydrogels were systematically evaluated against both *Escherichia coli* (*E. coli*) and *Staphylococcus aureus* (*S. aureus*) bacteria using standardized microbiological methods. For liquid-phase antibacterial assessment, bacterial suspensions (1 × 10^8^ CFU/mL) were prepared in nutrient broth and aliquoted into four treatment groups: (i) blank control, (ii) CMCS/SA hydrogel, (iii) CPSD hydrogel, and (iv) CPSD hydrogel + NIR. NIR irradiation was provided by an 808 nm CW diode laser (SLOC SDL-808) at 1.0 W cm^−2^ for 5 min, calibrated with a Thorlabs PM100D/S121C power meter. Following 4 h incubation at 37 °C with agitation (150 rpm), the optical density at 600 nm (OD_600_) was measured spectrophotometrically to quantify bacterial growth inhibition. The antibacterial efficiency was calculated as:

(3) $$Antibacterial\;efficiency\;\left(\%\right)=(1-\frac{{OD}_{n}}{{OD}_{0}})\times 100$$

*OD*_n_ represents the OD value of the Composite hydrogel, and *OD*_0_ denotes the OD value of the blank control group.

For solid-phase quantitative analysis, the standard plate count method was employed. Hydrogel samples (10 mm diameter) were placed in direct contact with bacterial lawns (1 × 10^6^ CFU) on LB agar plates. The experimental groups consisted of: (i) blank control (agar only), (ii) CMCS/SA hydrogel, (iii) CPSD hydrogel, and (iv) CPSD hydrogel + NIR. After 24 h incubation at 37 °C, the bacterial suspensions were serially diluted, plated on LB agar, and incubated for 18–24 h to count viable colonies. Bacterial suspensions were serially diluted in sterile PBS, plated on LBagar, and incubated for 18 h at 37 °C prior to colony counting. The antibacterial efficiency was calculated as:

(4) $$Antibacterial\;efficiency\;\left(\%\right)=(1-\frac{{CFU}_{n}}{{CFU}_{0}})\times 100$$

### 2.5. Cell Viability Assay of CPSD Hydrogels

#### 2.5.1. CCK8 Assay of CPSD Hydrogels

The cytocompatibility of CPSD hydrogels was evaluated using the CCK-8 assay with L929 mouse fibroblasts. Cells were seeded in 96-well plates at a density of 5 × 10^3^ cells/well in DMEM supplemented with 10% FBS and 1% penicillin-streptomycin, followed by 24 h incubation at 37 °C in 5% CO_2_ to allow for cell attachment. The culture medium was then replaced with 100 μL of fresh medium containing hydrogel extracts. After 24 h, 48 h and 72 h of exposure, 10 μL of CCK-8 reagent was added to each well and incubated for 2 h at 37 °C. The absorbance at 450 nm was measured using a microplate reader. Cell viability was calculated as follows:

(5) $$Viability\;\left(\%\right)=\left(\frac{{OD}_{sample}-{OD}_{positive}}{{OD}_{negative}-{OD}_{positive}}\;\right)\times 100\%$$

where *OD_sample_* is the absorbance of the treated group, *OD_negative_* is the absorbance of untreated cells, and *OD_positive_* is the absorbance of cells treated with 0.1% Triton X-100.

#### 2.5.2. Hemolysis Assay of CPSD Hydrogels

Fresh anticoagulated rat blood was obtained and diluted 10× with PBS (pH 7.4). CPSD and control hydrogels (n = 3) were incubated with 1 mL of diluted blood at 37 °C for 1 h. PBS and deionized water served as the negative and positive controls, respectively. After incubation, samples were centrifuged at 3000 rpm for 10 min, and the supernatant absorbance was measured at 540 nm. The hemolysis ratio (HR) was calculated as:

(6) $$Hemolysis\;Ratio\;=\left(\frac{{OD}_{sample}-{OD}_{negative}}{{OD}_{positive}-{OD}_{negative}}\;\right)\times 100\%$$

#### 2.5.3. Live/Dead Cell Staining of CPSD Hydrogels

To visualize cell viability, L929 cells were seeded into 24-well plates containing sterilized hydrogel samples and cultured for 12 h, 24 h, and 36 h. After incubation, cells were rinsed with PBS and stained with a Calcein-AM/Propidium Iodide (PI) Live/Dead Cell Imaging Kit (KeyGEN BioTECH, Nanjing, China) according to the manufacturer’s instructions. After 30 min incubation in the dark, cells were imaged using an inverted fluorescence microscope (Leica DMi8, Leica Microsystems, Wetzlar, Germany). Green fluorescence (Calcein-AM) indicates live cells, and red fluorescence (PI) indicates dead cells.

### 2.6. Antioxidant Activity Evaluation of CPSD Hydrogels

The free radical scavenging capacity of CPSD hydrogels was systematically evaluated using three complementary assays: PTIO (2-phenyl-4,4,5,5-tetramethylimidazoline-1-oxyl-3-oxide), ABTS (2,2′-azino-bis (3-ethylbenzothiazoline-6-sulfonic acid)), and DPPH (2,2-diphenyl-1-picrylhydrazyl). For PTIO radical scavenging assay, hydrogel samples were immersed in 1 mL of 100 μM PTIO solution (in 10 mM PBS, pH 7.4) and incubated at 37 °C protected from light. The decrease in PTIO radical concentration was monitored by measuring absorbance at 557 nm at different min using a UV-Vis spectrophotometer. ABTS radical cation scavenging activity was determined by mixing 100 μL hydrogel extract with 900 μL of ABTS^+^ solution (7 mM ABTS and 2.45 mM potassium persulfate, reacted for 16 h in dark), followed by absorbance measurement at 734 nm incubation at room temperature. For DPPH assay, 500 μL hydrogel extract was mixed with 500 μL of 100 μM DPPH methanolic solution and kept in dark for 30 min before measuring absorbance at 517 nm. The radical scavenging activity (%) was calculated using the following formula:

(7) $$Scavenging\;Ratio\;\left(\%\right)=\left(\frac{A_{control}-A_{sample}}{A_{control}}\;\right)\times 100\%$$

Among them, “*A_sample_*” represents the absorbance after the addition of free radicals and the sample, while “*A_control_*” represents the absorbance of the free radical solution.

### 2.7. Adhesion Test of Composite Hydrogels

Lap-shear adhesion was quantified using porcine skin as the substrate. Fresh dorsal skin was trimmed to ~1 mm subcutaneous fat, stored at 4 °C for ≤24 h, and equilibrated at 37 °C for 30 min before testing. Substrates were cleaned by ethanol/water sonication (2×, 10 min each) and N_2_-dried. Hydrogels (CPSD and CMCS/SA hydrogel) were cast to ~1.0 mm thickness; two coupons were overlapped with a 10 × 10 mm bonding area using a spacer. Adhesion tests were performed on a universal testing machine (Instron 5943, Instron, Norwood, MA, USA) with appropriate load cells (50–500 N) and pneumatic grips. A 0.1 N preload was applied for 2 min to ensure intimate contact (tissue kept moist with PBS). After 30 min at 37 °C, specimens were pulled to failure at 5 mm min^−1^ on a universal tester. For each substrate condition, three independent specimens were tested (n = 3).

### 2.8. WVTR Test of Composite Hydrogels

Water vapor transmission was measured on equilibrated hydrogel films (CPSD and CMCS/SA). Each specimen (effective exposed area ≈ 1 cm^2^) was sealed on a permeation cup containing deionized water, then incubated at 37 °C and 50% RH in a controlled chamber (Binder KBF 240, Binder World, Tuttlingen, Germany). Mass loss of the cup was recorded at fixed intervals using an analytical balance. Conditions mirror those reported in the draft and SI where WVTR results for both hydrogels are summarized. WVTR was computed as:

(8) $$WVTR\;\left(\%\right)=\left(\frac{∆m}{A\;\times \;∆t}\;\right)\times 100\%$$

where Δm  is the mass loss over the interval Δt, and A  is the exposed membrane area. All tests were conducted in triplicate (n = 3) unless otherwise stated.

### 2.9. Real Sensing Applications: Biosignal Acquisition, Processing, and Agreement Analysis

CPSD hydrogel electrodes were evaluated side-by-side with commercial Ag/AgCl electrodes under representative tasks including sit-to-stand, level walking, stair ascent/descent, and downhill walking, with each task comprising at least three repeated trials. CPSD pads (Ø 20–25 mm, thickness ~1.0 mm) were placed over the vastus lateralis and tibialis anterior muscles, secured with medical tape. Lead wires were strain-relieved along the exoskeleton frame with slack at joints to minimize motion artifacts. Signals were acquired using an ADS1292-based front-end, with sampling frequency set at 1000 Hz, band-pass filtered at 20–500 Hz, and a 50 Hz notch filter applied to remove power-line noise. The inter-electrode distance was maintained at approximately 20 mm, and skin was prepared by shaving and wiping with alcohol to ensure low impedance contact. The sEMG signals were DC-removed, full-wave rectified, and linear-enveloped following established recommendations. Event markers were synchronized with exoskeleton motion phases, and analysis windows were aligned to steady-state or peak-activation epochs for power spectral density (PSD) and envelope feature extraction.

### 2.10. Statistical Analysis

All quantitative data are presented as mean ± standard deviation (SD) from at least three independent experiments. Statistical comparisons between two groups were performed using an unpaired two-tailed Student’s *t*-test. For multiple group comparisons, one-way analysis of variance (ANOVA) followed by Tukey’s post hoc test was used. A *p*-value less than 0.05 was considered statistically significant.

## 3. Results

### 3.1. Synthesis and Characterization of CPSD Composite Hydrogel

**Figure 1:**
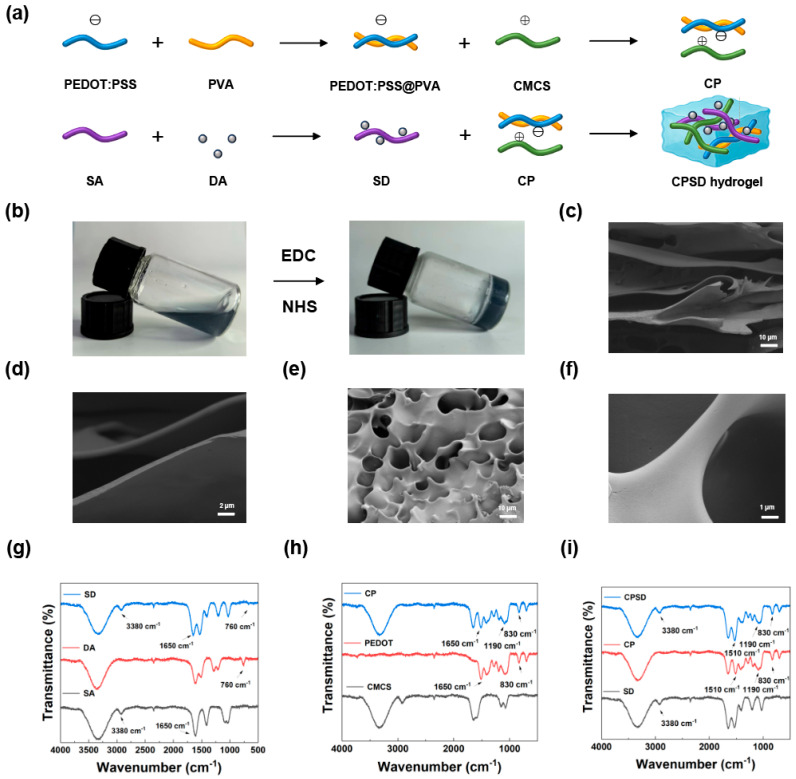
Preparation and characterization of CPSD composite hydrogels. (**a**) Schematic diagram of the CPSD hydrogel fabrication process. (**b**) Sol–gel transition behavior of the CPSD precursor solution. (**c**,**d**) SEM images of CMCS/SA hydrogel at low and high magnifications, respectively. (**e**,**f**) SEM images of CPSD hydrogel at low and high magnifications, respectively. (**g**) FTIR spectra of SD, DA, and SA, showing characteristic peaks indicating successful grafting. (**h**) FTIR spectra of CP, CMCS, and PEDOT:PSS, confirming physical interactions and component retention. (**i**) FTIR spectra of CPSD, CP, and SA, verifying the formation of the composite network.

To elucidate the structural features of the CPSD composite hydrogel, a series of morphological and spectroscopic characterizations were conducted. As illustrated in Figure 1a, the CPSD hydrogel was fabricated via a two-step hybridization strategy: (1) electrostatic complexation between positively charged CMCS and negatively charged PEDOT:PSS to construct a conductive polymeric backbone, and (2) DA grafting onto SA to enhance tissue adhesion. Subsequent EDC/NHS-mediated amidation enabled rapid in situ gelation at room temperature, forming a self-supporting hydrogel with integrated conductivity and bioadhesion (Figure 1b).

SEM was employed to analyze the internal microstructure of freeze-dried hydrogel samples. The CMCS/SA control hydrogel exhibited a relatively loose and disordered porous morphology (Figure 1c,d), whereas the CPSD hydrogel presented a more compact and homogeneous porous network with reduced pore size (Figure 1e,f, for a wider field of view). This densification can be attributed to π-π stacking and hydrogen bonding interactions between PEDOT:PSS and the polymer chains, leading to a more cohesive and structurally robust network. The chemical structure of the hydrogels was further investigated using FTIR spectroscopy. As shown in Figure 1g, characteristic peaks of SA were observed at ~1600 cm^−1^ and ~1410 cm^−1^, corresponding to asymmetric and symmetric stretching vibrations of carboxylate groups, respectively. After dopamine grafting, the resulting SA-DA (SD) exhibited additional absorption bands at ~1650 cm^−1^ (amide I) and ~1540 cm^−1^, confirming successful amide bond formation between SA and DA [37]. In Figure 1h, the CP hydrogel (CMCS combined with PEDOT:PSS) displayed typical PEDOT absorption bands, including C=C symmetric stretching (~1510 cm^−1^) and C-O-C stretching (~1190 cm^−1^) [38], indicating the stable incorporation of the conductive polymer. The final CPSD hydrogel spectrum (Figure 1i) exhibited integrated features from both SD and CP precursors. Notably, the presence of amide I and II bands and the PEDOT-related peaks collectively confirmed the formation of a dual-functional network with both adhesion and electrical conduction capabilities.

To further corroborate these structural findings, complementary mechanical/electrical tests (Figure S3) were conducted. Under uniaxial compression (Figure S3a), CPSD displayed a J-shaped stress–strain profile and reached ~20 kPa at high strain, exceeding CMCS/SA. Wet-tissue lap-shear (Figure S3c) also increased from ~3 kPa (CMCS/SA) to ~10 kPa (CPSD), indicating stronger interfacial adhesion. Four-probe measurements (Figure S3b) showed a higher bulk conductivity for CPSD (1.5 S m^−1^) versus CMCS/SA (0.3 S m^−1^). Importantly, in situ tensile tests revealed a strain-dependent yet usable conductivity (Figure S3d), decreasing from ~1.5 S m^−1^ at 0% to ~0.6 S m^−1^ at 40% strain, supporting both stable electrical function and intrinsic mechanosensing in the deformation range relevant to soft-tissue motion.

Together, these results demonstrate the successful fabrication of CPSD hydrogels with a well-defined porous structure and chemically crosslinked architecture, which lay the foundation for their multifunctionality in bioelectronic and tissue-interfacing applications.

### 3.2. Fundamental Properties of CPSD Hydrogel

#### 3.2.1. The Swelling, Degradation and WVTR Properties of CPSD

**Figure 2:**
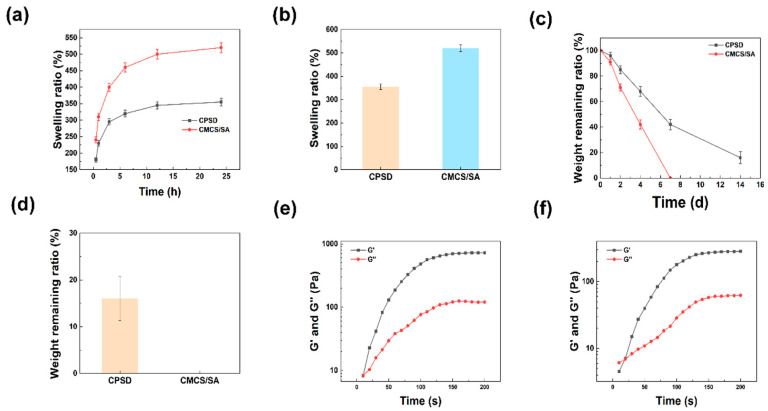
Fundamental properties of the CPSD composite hydrogel: (**a**) swelling curves of CPSD and CMCS/SA hydrogels; (**b**) quantitative swelling statistics of CPSD and CMCS/SA; (**c**) degradation curves of CPSD and CMCS/SA; (**d**) quantitative degradation statistics of CPSD and CMCS/SA; (**e**) rheological profile of CPSD hydrogel; (**f**) rheological profile of CMCS/SA hydrogel.

To assess the hydration behavior and moisture-management capacity of CPSD hydrogels, their swelling, degradation, and water-vapor transmission properties were systematically characterized. As shown in Figure 2a,b, the CPSD hydrogel exhibited a significantly lower swelling ratio than the CMCS/SA system, with equilibrium swelling reaching ~350% compared to over 500% for CMCS/SA. This reduced water uptake can be attributed to the introduction of PEDOT:PSS and dopamine, which increase the crosslinking density and hydrophobic interactions within the CPSD network, thereby restricting excessive hydration. From a functional perspective, this moderate swelling capacity is beneficial for exudate management in biomedical applications, as it maintains a moist microenvironment while preventing over-swelling that may compromise the structural stability of the dressing.

The degradation behavior further highlights the stabilizing effect of PEDOT:PSS and dopamine (Figure 2c,d). While the CMCS/SA hydrogel completely degraded within 7 days, CPSD retained a small but measurable fraction even after 14 days. This extended degradation profile suggests that CPSD possesses enhanced resistance against hydrolytic breakdown, which is advantageous for applications requiring longer-term coverage or sustained therapeutic effects. The controlled degradation also implies that the incorporation of conductive polymers may effectively modulate the balance between biodegradability and mechanical durability. Moreover, water vapor transmission measurements (Figure S4) showed that CMCS/SA exhibited a WVTR of ~142 g·m^−2^·h^−1^, while CPSD displayed a reduced but still appropriate value of ~75 g·m^−2^·h^−1^, suggesting balanced moisture retention and permeability. Collectively, these results highlight that CPSD hydrogels not only provide superior mechanical strength, conductivity, and adhesion but also maintain suitable vapor permeability, underscoring their potential in bioelectronic and wearable applications.

The physicochemical and functional analyses collectively demonstrate that the CPSD hydrogel combines moderate swelling, stable degradation, and appropriate water-vapor transmission, which jointly ensure moisture balance and mechanical stability under skin-contact conditions. Such a combination is essential for long-term biocompatibility and comfort during exoskeleton-assisted motion.

#### 3.2.2. The Rheological and Mechanical Properties of CPSD

Following the evaluation of hydration-related properties, the rheological and mechanical behaviors were further examined to elucidate the structural robustness of the CPSD network. Rheological analysis confirmed the rapid gelation and robust mechanical properties of CPSD. As shown in Figure 2e, CPSD underwent sol–gel transition within 10 s, reaching a maximum storage modulus (G′) of ~724 Pa. In contrast, CMCS/SA (Figure 2f) required approximately 20 s to achieve gelation, and its maximum G′ plateaued at ~283 Pa. The higher G′ of CPSD indicates a stronger elastic network, likely resulting from synergistic crosslinking between the polysaccharide backbone and the π–π interactions of PEDOT:PSS and dopamine. Such fast gelation kinetics and enhanced stiffness are critical for in situ applications, particularly where rapid gel formation and mechanical stability are necessary to adapt to dynamic biological environments.

In addition, the fundamental physicochemical properties of the hydrogels were further evaluated (Figures S3 and S4). The compressive curves (Figure S3a) showed that the CPSD hydrogel possessed a compressive modulus of ~15 kPa with a maximum stress of ~20 kPa, which was markedly higher than that of CMCS/SA (~3 kPa and ~4 kPa, respectively). Conductivity measurements (Figure S3b) indicated that CPSD reached ~1.6 S·m^−1^, compared with ~0.38 S·m^−1^ for CMCS/SA, confirming the effective incorporation of the conductive network. Lap-shear tests (Figure S3c) further revealed that the adhesion strength of CPSD (~9.5 kPa) was almost fourfold that of CMCS/SA (~2.6 kPa), attributable to the catechol-mediated interfacial bonding and reinforced polymer network.

Taken together, these results demonstrate that CPSD hydrogel integrates favorable swelling, slower degradation, and superior rheological behavior compared to CMCS/SA. The balanced physicochemical properties underscore the potential of CPSD as a multifunctional material platform, particularly in scenarios that demand both stability and bioadaptability.

Overall, the hydration control, degradation resistance, and moisture permeability of CPSD hydrogels collectively ensure structural stability and skin adaptability. Although long-term hydrolysis under dynamic mechanical stress was not assessed, the balanced physicochemical performance supports their potential as reliable biointerfaces for motion sensing and rehabilitation applications.

### 3.3. Antioxidant Properties of CPSD Composite Hydrogel

**Figure 3:**
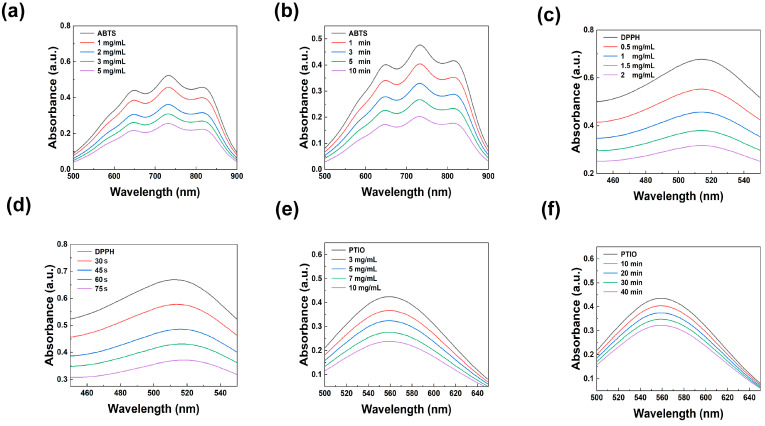
UV–vis spectra illustrating the antioxidant capacity of CPSD hydrogel extracts: (**a**,**b**) ABTS radical scavenging at different concentrations (10 min) and at 1 mg/mL (3 min) over varying time intervals; (**c**,**d**) DPPH radical scavenging at different concentrations (30 s) and at 1 mg/mL over varying time intervals; (**e**,**f**) PTIO radical scavenging at different concentrations (10 min) and at 5 mg/mL over varying time intervals.

In the exoskeleton-assisted rehabilitation environment, mechanical stress is prone to cause the wound tissue to produce a large amount of reactive oxygen species (ROS). These free radicals not only damage the cell structure but also significantly delay the tissue regeneration process. Therefore, constructing intelligent hydrogel materials with effective free radical scavenging capabilities has become a key strategy for improving the quality and rate of wound healing. As shown in Figure 3a,b, CPSD hydrogel extracts display clear ABTS scavenging with a representative value of 57.56% at 2 mg mL^−1^ (10 min). Figure 3c,d shows similarly strong and fast activity toward DPPH, reaching 53.18% at 2 mg mL^−1^ (30 s). For PTIO, Figure 3e,f reveals a relatively lower but still meaningful quenching capacity, exemplified by 43.63% at 10 mg mL^−1^ (10 min) (Figure S5). Across the concentration-series panels (a, c, e) and the time-course panels (b, d, f), the trends are dose- and time-dependent, indicating both rapid and sustained radical removal.

Therefore, the outstanding ROS scavenging ability of CPSD hydrogel may be attributed to the synergistic effect of the phenolic hydroxyl groups in the DA molecular structure and the conjugated π system of PEDOT:PSS. This not only enhances the electron transfer efficiency but also endows the material with a broad-spectrum free radical response capability, providing the possibility for its application in wound regulation in complex inflammatory microenvironments.

Collectively, the ABTS, DPPH, and PTIO assays demonstrate that CPSD exhibits a rapid, dose-dependent, and sustained free-radical-scavenging capability. This capacity enables the hydrogel to form a stable antioxidative buffer within a short time, thereby mitigating oxidative stress that could interfere with tissue reconstruction and interfacial adhesion or electrical coupling. It should be noted that these results were obtained from extracellular chemical models and do not fully represent the complex biochemical environment of body fluids or long-term mechanical loading; further evaluation under physiologically relevant conditions is warranted. Nevertheless, the current findings confirm that CPSD can effectively regulate the interfacial redox state in the high-stress, ROS-rich conditions of exoskeleton-assisted rehabilitation, providing a more reliable microenvironment for biosignal acquisition and wound management.

### 3.4. Antibacterial Properties of CPSD Composite Hydrogel

**Figure 4:**
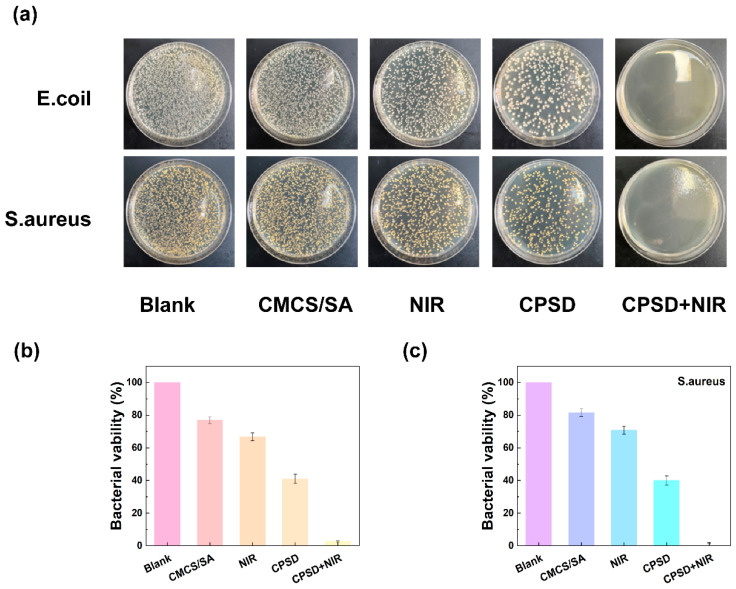
The plate-counting method graph shows the antibacterial ability of the CPSD hydrogel extract: (**a**) Representative colony photographs of *E. coli* and *S. aureus* cultured with Blank, CMCS/SA, NIR, CPSD, and CPSD + NIR groups. (**b**) Quantitative antibacterial analysis against *E. coli* in the different groups. (**c**) Quantitative antibacterial analysis against *S. aureus* in the different groups.

During the process of lower extremity exoskeleton-assisted rehabilitation, sweat and environmental exposure are prone to cause wound contamination. Therefore, smart materials with excellent antibacterial properties are the key to promoting the development of therapeutic systems. To systematically evaluate the antibacterial effect of CPSD hydrogel, solid plate count, were adopted, respectively, in the *E. coli* and *S. aureus* models (Figure 4a). The solid antibacterial results showed that compared with the CMCS/SA control group, CPSD hydrogel had significant antibacterial effects on both *E. coli* and *S. aureus*. The antibacterial rate of *E. coli* was 83.7% ± 3.2% (about 0.79 log_10_ reduction), and that of *S. aureus* was 78.9% ± 2.6% (about 0.68 log_10_ reduction) (Figure 4b). When combined with 808 nm NIR laser irradiation (1.0 W/cm^2^, 5 min), the antibacterial performance of CPSD hydrogel was further enhanced, and the antibacterial rates of *E. coli* and *S. aureus* reached 95.2% ± 1.9% and 91.6% ± 2.4% (Figure 3c), respectively. Figure S6 presents the antibacterial test results of the control groups containing only SA-DA or only PEDOT:PSS. The results show that neither SA-DA alone nor PEDOT:PSS alone exhibited any antibacterial effect. This indicates that it has good potential for photothermal assisted antibacterial properties. Solid plate counting further verified the above results.

The outstanding antibacterial performance of CPSD hydrogel can be attributed to its multiple synergistic mechanisms: on the one hand, CMCS themselves have natural cationic properties and can destroy bacterial cell membranes through charge action [39]; On the other hand, the photothermal synergistic effect of DA and PEDOT:PSS endows the hydrogel with excellent photothermal properties, thereby achieving the “photothermal sterilization” effect and further enhancing the antibacterial effect [40].

The strong antibacterial activity and ROS-scavenging ability of CPSD contribute to maintaining a clean and redox-balanced microenvironment at the skin–hydrogel interface. While the antibacterial evaluation was limited to two strains in vitro, these results highlight the material’s potential to reduce infection and inflammation risks during prolonged skin contact.

### 3.5. Biocompatibility Performance of CPSD Composite Hydrogel

**Figure 5:**
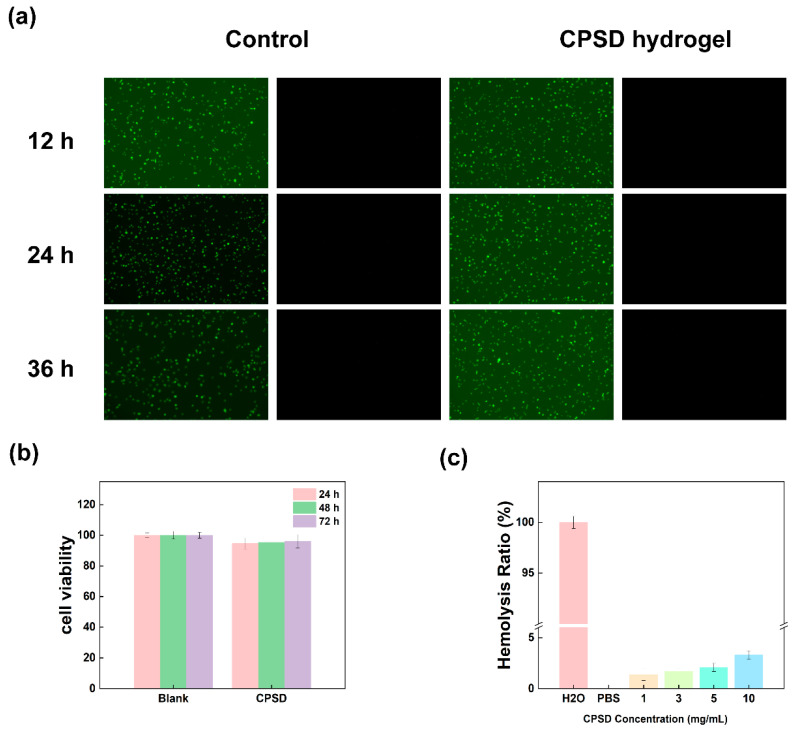
Live cell staining and CCK8 assays demonstrated the cell safety capability of the CPSD hydrogel extract: (**a**) Fluorescence images of L929 fibroblasts co-cultured with the extract of CPSD hydrogel and the control group at 12, 24, and 36 h. (**b**) CCK-8 assay results of L929 fibroblasts co-cultured with the extract of CPSD hydrogel and the control group at 12, 24, and 36 h. (**c**) Hemolysis rates of CPSD hydrogel at concentrations of 1, 3, 5, and 10 mg/mL.

Biocompatibility is a fundamental requirement for the application of hydrogel materials in tissue repair and wearable medical devices. Especially for systems that adhere to the skin or wounds for a long time, it is necessary to ensure their safety at the cellular level and in the blood system. In the CCK-8 experiment, as shown in Figure 5a,b, the L929 mouse fibroblast model was used, and the effect of hydrogel extract on cell activity was analyzed by the CCK-8 method. The results showed that after 24 h, 48 h and 72 h treatment, neither the CMCS/SA nor the CPSD hydrogels significantly inhibited cell proliferation. Especially in the CPSD group, the cell viability remained at 96.4% ± 2.1% at 72 h, showing no statistical difference from the control group, indicating that the hydrogel still maintained excellent cytocompatibility under long-term exposure conditions. This is attributed to the biodegradable properties of the natural polymers CMCS and SA in the hydrogel composition. Meanwhile, the introduction of PEDOT:PSS did not induce significant cytotoxicity, indicating that its electrical functional modules do not interfere with the cellular metabolic process. To further confirm the biological safety of CPSD hydrogel in the blood environment, its hemolysis rate was determined (Figure 5c). According to ASTM standards, a hemolysis rate of less than 5% can be considered to have good blood compatibility (Figure S7). The experimental results show that the hemolysis rates of the CMCS/SA and CPSD groups are 2.3% and 2.6%, respectively, both far below the safety threshold (Figure S8). This indicates that they do not trigger hemolysis reactions when in short-term contact with wound exudate or blood, and have the basic conditions for use in trauma application or implantation systems [41]. Subsequently, imaging analysis was conducted on the survival of L929 cells after treatment with hydrogel extract at 12 h, 24 h, and 36 h. Green fluorescence represents living cells, while red fluorescence represents dead cells. Observations from the images at the three time points show that the CPSD group has almost no red fluorescence signal, only presenting a large amount of green fluorescence. The cell morphology is intact and well extended, and there is no significant difference from the CMCS/SA group, further confirming its low toxicity and good ability to support cell adhesion/expansion. Therefore, CPSD hydrogels do not exhibit toxic or side effects at the cellular level and have good compatibility in the blood environment, meeting the safety requirements for use as interface materials in chronic wound management and wearable devices.

The in vitro and in vivo results confirm that CPSD hydrogels exhibit excellent cytocompatibility and tissue safety without observable toxicity. Although long-term degradation behavior in vivo requires further monitoring, the current findings support their suitability for chronic wearable or implantable bioelectronic applications.

### 3.6. Application of CPSD Composite Hydrogel Lower Limb Exoskeleton

**Figure 6:**
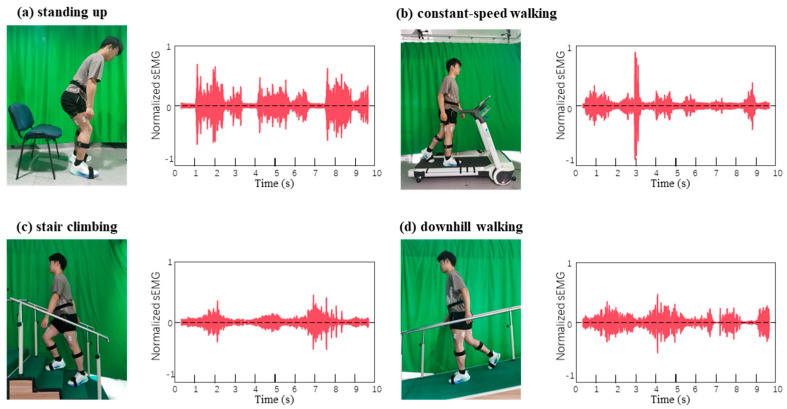
Motion photos and data collection demonstrate the motion detection capability of the CPSD hydrogel: (**a**) shows the collected electromyographic changes in the subjects from sitting to standing. (**b**) shows the collected electromyographic changes in the subjects walking on flat ground. (**c**) shows the collected electromyographic changes in the subjects climbing steps. (**d**) shows the collected electromyographic changes in the subjects going down a slope.

The integration of the CPSD hydrogel into a hip-actuated lower-limb exoskeleton highlights its potential as a soft bioelectronic interface for motion sensing and rehabilitation. As shown in Figure 6, the hydrogel electrodes provided stable and high-fidelity electromyographic (EMG) recordings across different functional tasks [42]. During the sit-to-stand transition (Figure 6a), sharp EMG bursts were observed, reflecting the rapid recruitment of the rectus femoris to overcome initial inertia. In level-ground walking (Figure 6b), the CPSD electrodes captured rhythmic and periodic activity that could be clearly segmented into gait cycles, enabling reliable intent detection and phase recognition. When subjects ascended stairs (Figure 6c), EMG amplitudes increased markedly, consistent with the higher antigravity demand, whereas downhill walking (Figure 6d) displayed moderate but frequent fluctuations, indicative of eccentric muscle control [43].

These task-specific signal characteristics demonstrate that CPSD hydrogel electrodes can effectively discriminate between dynamic locomotor states. Compared with conventional Ag/AgCl electrodes, the CPSD interface offered not only comparable signal quality but also superior conformability, biocompatibility, and long-term stability, thereby reducing motion artifacts during complex movements. Importantly, the clear phase-dependent patterns extracted from the rectus femoris enabled real-time closed-loop control of the exoskeleton [44]. By linking EMG amplitude thresholds with motor torque assistance, the system achieved seamless transitions between flexion, extension, and stance phases, ensuring both responsiveness and user safety. It is worth noting that signal quality was influenced by skin–electrode motion, sweat-induced contact-impedance shifts, and cable micro-movement. Mitigation included skin preparation, standardized placement, and lead strain-relief along the exoskeleton. Consistency between CPSD and clinical Ag/AgCl electrodes was further quantified by a Bland–Altman analysis of the surface-EMG power spectra, showing a negligible bias and narrow 95% limits of agreement (−5.9% to +4.7%) across pooled tasks/participants (Figure S9), indicating practical interchangeability for spectral estimates.

Beyond motion intention detection, the quantitative EMG indices (RMS, MAV, iEMG) further revealed reduced muscle activation under exoskeleton assistance, confirming effective torque sharing. Conversely, in participants with reduced muscle strength, longitudinal EMG monitoring suggested progressive enhancement in muscle engagement, underscoring the rehabilitative potential of this approach. Taken together, these findings highlight that CPSD hydrogels serve as multifunctional electrodes capable of bridging biological signals with robotic actuation, thus enabling both precise motor assistance and quantitative evaluation of rehabilitation progress.

## 4. Discussion

In this study, we successfully developed a multifunctional CPSD hydrogel via a two-step hybrid strategy combining electrostatic self-assembly and EDC/NHS-mediated covalent crosslinking. The integration of conductive PEDOT:PSS and dopamine-functionalized alginate into a CMCS-based matrix endowed the hydrogel with a tissue-conformal porous architecture and robust mechanical integrity. Comprehensive characterization demonstrated that CPSD hydrogels exhibit tunable swelling and degradation profiles, favorable viscoelasticity, and repeatable photothermal responses under NIR stimulation.

Importantly, CPSD hydrogels displayed superior antioxidant capacity in DPPH, ABTS, and PTIO radical scavenging assays, as well as potent antibacterial effects against both Gram-negative and Gram-positive strains, particularly under NIR exposure. In vitro cytocompatibility and hemocompatibility assessments confirmed excellent biocompatibility, as evidenced by high cell viability, negligible hemolysis, and minimal cytotoxicity over 48 h. Notably, when applied in a wearable lower-limb exoskeleton system, CPSD hydrogels adhered stably to the joint area during motion and generated reliable strain signals for real-time monitoring of joint activity.

A focused literature comparison (Table S1) [28,34,45,46,47,48,49,50] situates these findings. Recent conductive or adhesive hydrogels often emphasize strain sensing and self-adhesion but do not report standardized wet-tissue lap-shear on porcine skin or WVTR Systems incorporating antibacterial components such as ZnO or polypyrrole demonstrate microbicidal effects yet still lack WVTR or standardized wet-adhesion tests in many cases. Electrode agreement analyses against Ag/AgCl are common in textile or other dry electrodes but remain limited for hydrogel interfaces. WVTR methodologies are available and highlight the importance of moisture balance at the skin interface. In contrast, the present work consolidates wet-tissue adhesion under a defined protocol, WVTR under controlled temperature/humidity, dual antioxidative and antibacterial functions with optional NIR enhancement, and Bland–Altman agreement with Ag/AgCl (Figure S9), together with strain-dependent yet usable conductivity during deformation (Figure S3d). This combination addresses sensing, protection, and biointerface reliability in a single material platform.

These results demonstrate that CPSD hydrogels achieve synergistic integration of bioadhesion, conductivity, antioxidative and antibacterial capacity, and stable signal transduction. Such multifunctionality enables the material to serve as both a protective dressing and a real-time biosensing interface in dynamic rehabilitation environments.

## 5. Conclusions

Taken together, this work presents CPSD hydrogel as a promising bioelectronic interface material that bridges soft tissue healing with active motion sensing. The multifunctional integration of adhesion, conductivity, antioxidation, and strain responsiveness provides a unified platform for next-generation wearable rehabilitation and mobile healthcare monitoring. Future work will focus on optimizing hydrogel–tissue coupling for long-term performance and expanding the sensing modalities to achieve closed-loop therapeutic feedback.

## Data Availability

The original contributions presented in this study are included in this article. Further inquiries can be directed to the corresponding author.

## Supplementary Materials

The following supporting information can be downloaded at: https://www.mdpi.com/article/10.3390/s25216727/s1, Figure S1: SEM images of CPSD and CMCS/SA hydrogels; Figure S2: Cross-sectional SEM of CPSD hydrogel. Left: overview (scale bar 400 μm). Right: higher magnification highlighting interlayer bridges and open pores (scale bar 50 μm); Figure S3: Mechanical, electrical, and adhesion characterization of CMCS/SA and CPSD hydrogels. (a) Uniaxial compression stress–strain curves of hydrogel cylinders. (b) Bulk electrical conductivity determined by the four-probe method on hydrated slabs. (c) Lap-shear stress–strain curves for wet-tissue adhesion on porcine skin using a modified ASTM F2255 protocol. (d) Electrical conductivity of CPSD hydrogels as a function of tensile strain; Figure S4: Water vapor transmission rate (WVTR) of CPSD and CMCS/SA hydrogels; Figure S5: Statistical analysis of the radical scavenging capacity of CPSD hydrogel extracts: (a,b) ABTS radical scavenging efficiency at different concentrations (3 min) and at 1 mg/mL (3 min); (c,d) DPPH radical scavenging efficiency at different concentrations (30 s) and at 1 mg/mL over varying time intervals; (e,f) PTIO radical scavenging efficiency at different concentrations (10 min) and at 5 mg/mL over varying time intervals; Figure S6: Antibacterial assay of control groups containing only SA-DA or only PEDOT:PSS: (a) plate photographs of SA-DA co-cultured with *E. coli*; (b) SA-DA co-cultured with *S. aureus*; (c) PEDOT:PSS co-cultured with *E. coli*; (d) PEDOT:PSS co-cultured with *S. aureus*; Figure S7: Hemolysis images at different concentrations, with H_2_O as the positive control and PBS as the negative control; Figure S8: Hemolysis rate of CMCS/SA hydrogel at 10 mg/mL; Figure S9: Bland–Altman consistency analysis of surface electromyographic power spectra of CPSD hydrogel electrodes and Ag/AgCl electrodes; Table S1: Focused literature comparison highlighting dimensions covered by this work (√ = reported; × = not reported).
