# Supplementary material for: Biointegrated Conductive Hydrogel for Real-Time Motion Sensing in Exoskeleton-Assisted Lower-Limb Rehabilitation
Source: Sensors (Basel). 2025 Nov 3;25(21):6727. doi: 10.3390/s25216727 (PMC12610655; doi:10.3390/s25216727)
Supplement: Supplementary file 1 [file sensors-25-06727-s001.zip › sensors-3894207-supplementary.pdf]

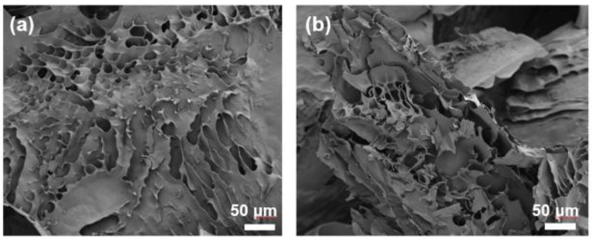

Figure S1. SEM images of CPSD and CMCS/SA hydrogels.

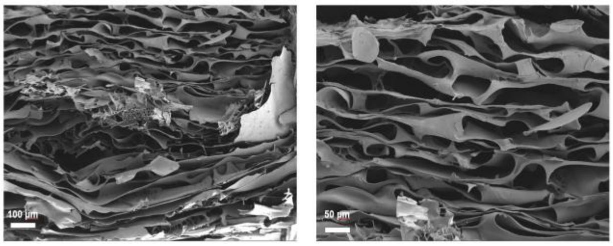

Figure S2. Cross-sectional SEM of CPSD hydrogel. Left: overview (scale bar 400 μm). Right: higher magnification highlighting interlayer bridges and open pores (scale bar 50 μm).

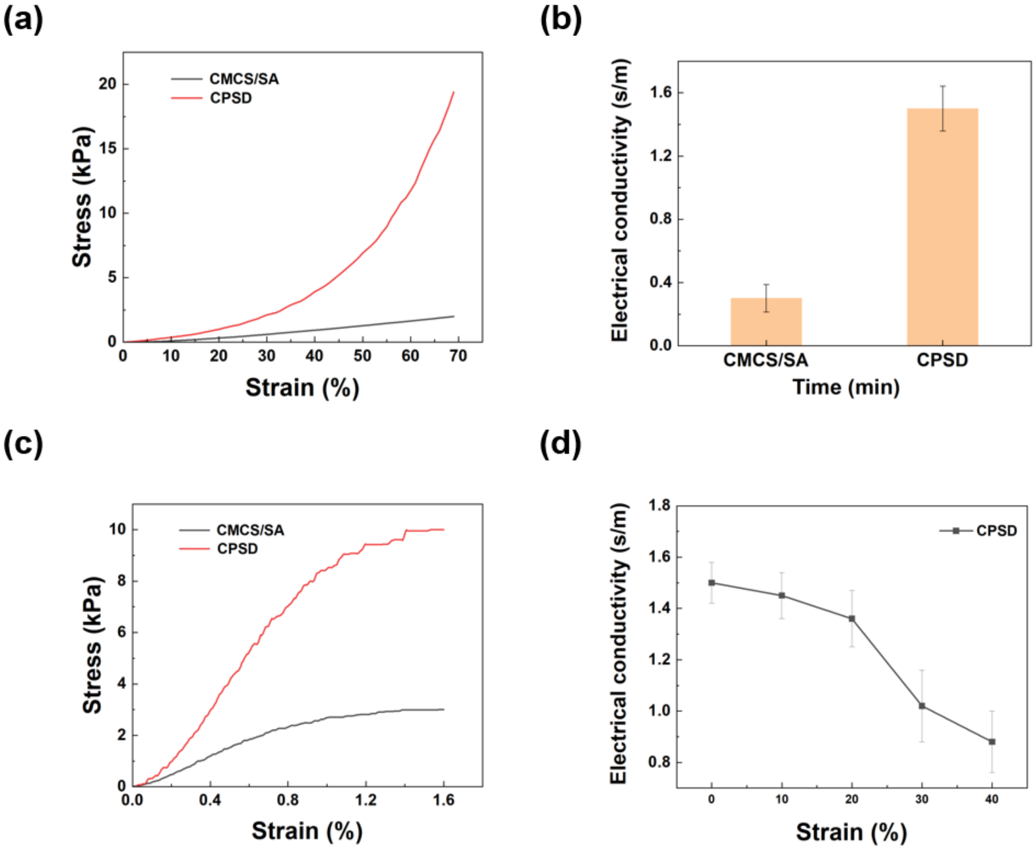

Figure S3. Mechanical, electrical, and adhesion characterization of CMCS/SA and CPSD hydrogels. (a) Uniaxial compression stress–strain curves of hydrogel cylinders. (b) Bulk electrical conductivity determined by the four-probe method on hydrated slabs. (c) Lap-shear stress−strain curves for wet-tissue adhesion on porcine skin using a modified ASTM F2255 protocol (d) Electrical conductivity of CPSD hydrogels as a function of tensile strain.

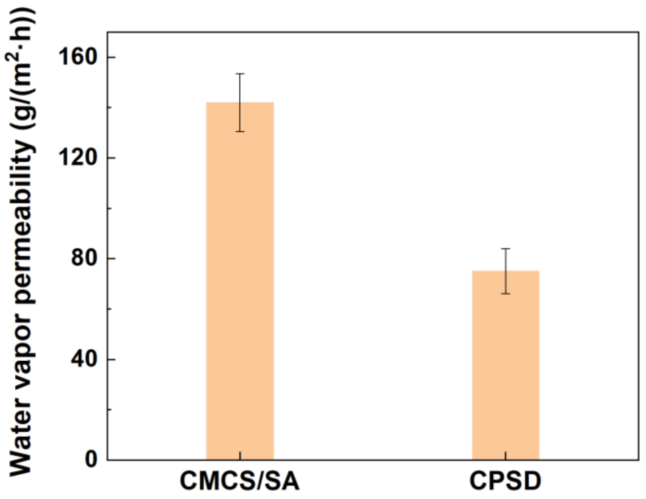

Figure S4. Water vapor transmission rate (WVTR) of CPSD and CMCS/SA hydrogels.

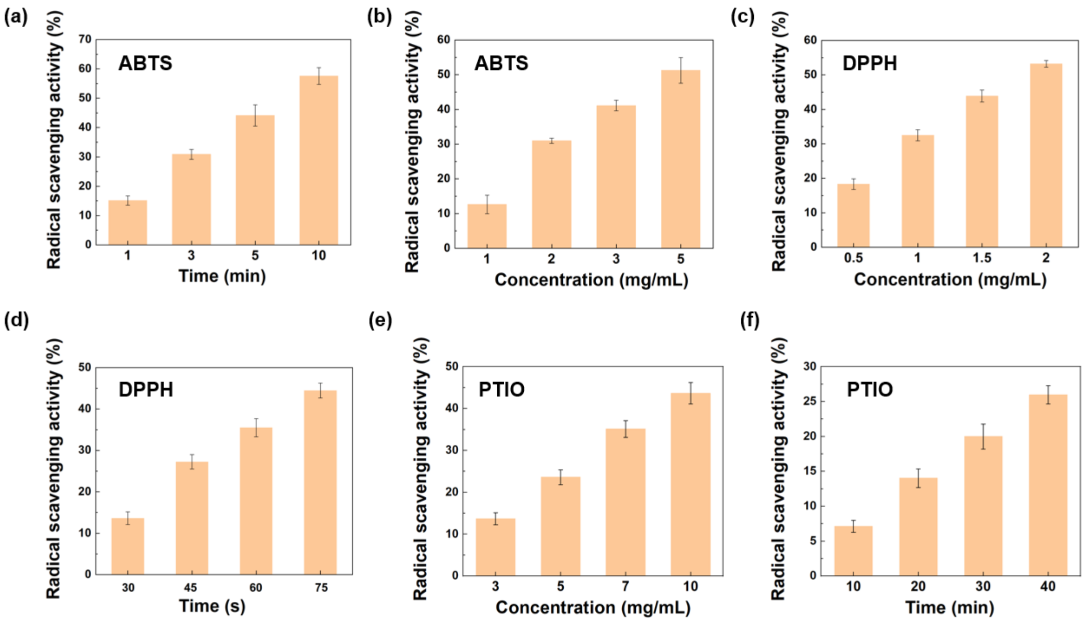

Figure S5. Statistical analysis of the radical scavenging capacity of CPSD hydrogel extracts: (a,b) ABTS radical scavenging efficiency at different concentrations (3 min)

and at 1 mg/mL (3 min); (c,d) DPPH radical scavenging efficiency at different concentrations (30 s) and at 1 mg/mL over varying time intervals; (e,f) PTIO radical scavenging efficiency at different concentrations (10 min) and at 5 mg/mL over varying time intervals.

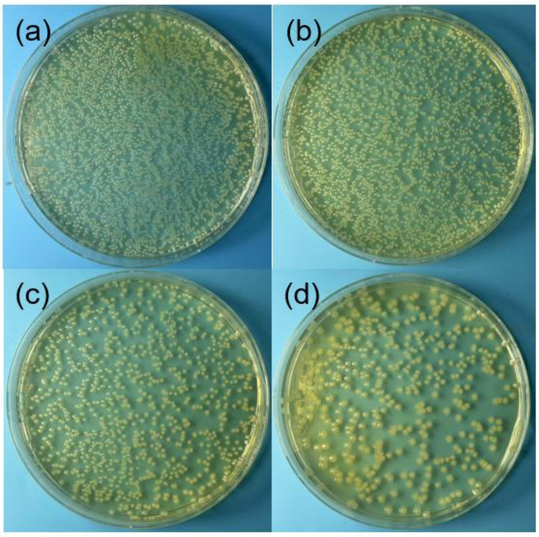

Figure S6. Antibacterial assay of control groups containing only SA-DA or only PEDOT:PSS: (a) plate photographs of SA-DA co-cultured with *E. coli*; (b) SA-DA co-cultured with *S. aureus*; (c) PEDOT:PSS co-cultured with *E. coli*; (d) PEDOT:PSS co-cultured with *S. aureus*.

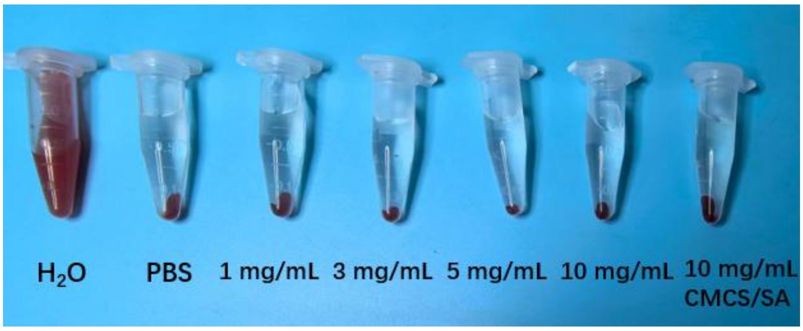

Figure S7. Hemolysis images at different concentrations, with H_2O as the positive control and PBS as the negative control.

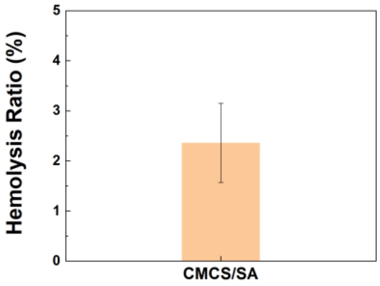

Figure S8. Hemolysis rate of CMCS/SA hydrogel at 10 mg/mL

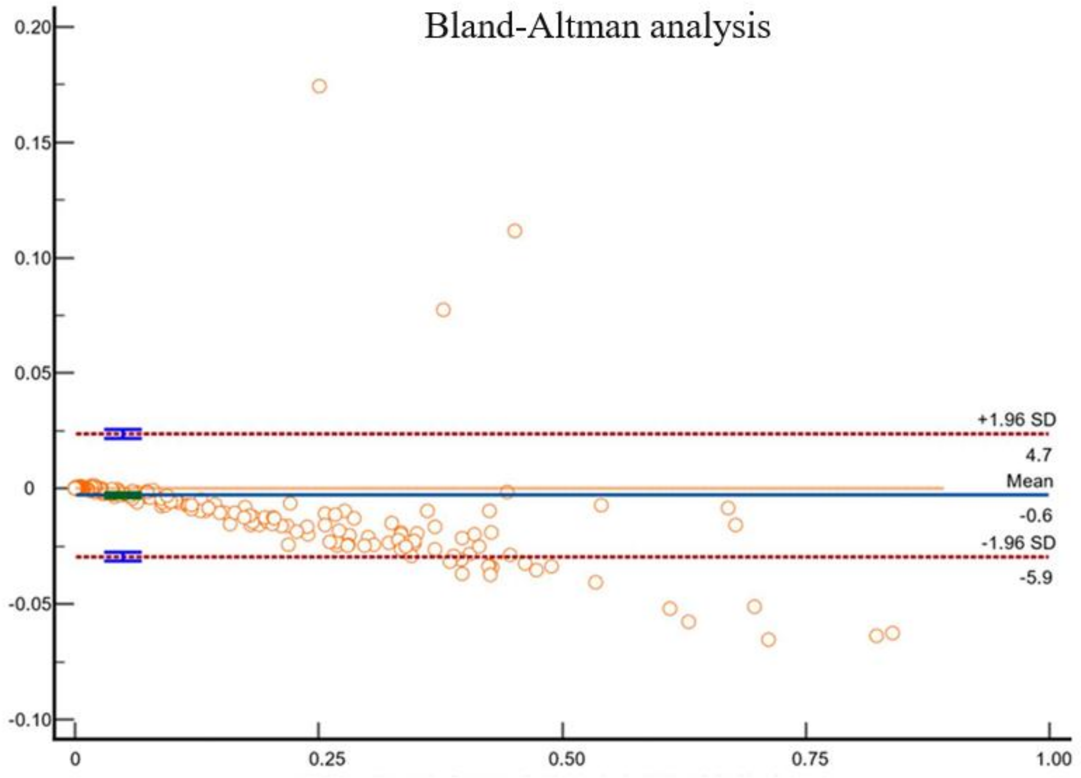

Figure S9. Bland–Altman consistency analysis of surface electromyographic power spectra of CPSD hydrogel electrodes and Ag/AgCl electrodes

Table S1. Focused literature comparison highlighting dimensions covered by this work

(√ = reported; × = not reported.)

Cao et al., Int. J. Biol. Macromol. (2024). DOI: 10.1016/j.ijbiomac.2024.136581	×	×	×	×	×
Liao et al., Molecules (2024) 29:5728. DOI: 10.3390/molecules29235728	×	×	×	√	×
Qin et al., ACS Omega (2024) 9:126202. DOI: 10.1021/acsomega.4c01860	×	×	×	√	×
Chen et al., ACS Biomater. Sci. Eng. (2022) 8:2471–2482. DOI: 10.1021/acsbiomaterials.1c01630	×	×	×	√	×

Sun et al., RSC Adv. (2023) 13:5762 – 5769. DOI: 10.1039/D3RA00546A	×	×	×	√	×
Lam et al., J. Rehabil. Assist. Technol. Eng. (2022) 9:1 – 18. DOI: 10.1177/20556683211061995	×	×	×	×	×
Kim et al., Polymers (2020) 12:2406. DOI: 10.3390/polym12102406	×	×	×	×	×
Tyuftin et al., Sustainability (2022) 14:9018. DOI: 10.3390/su14159018	×	√	×	×	×
